# Sulfonation, an underexploited area: from skeletal development to infectious diseases and cancer

**DOI:** 10.18632/oncotarget.10046

**Published:** 2016-06-14

**Authors:** Ada W.Y. Leung, Ian Backstrom, Marcel B. Bally

**Affiliations:** ^1^ Experimental Therapeutics, BC Cancer Research Centre, Vancouver, BC, Canada; ^2^ Department of Pathology and Laboratory Medicine, University of British Columbia, Vancouver, BC, Canada; ^3^ Faculty of Pharmaceutical Sciences, University of British Columbia, Vancouver, BC, Canada; ^4^ Centre for Drug Research and Development, Vancouver, BC, Canada

**Keywords:** sulfonation, PAPSS, sulfotransferases, heparan sulfate, tyrosine sulfation

## Abstract

Sulfonation is one of the most abundant cellular reactions modifying a wide range of xenobiotics as well as endogenous molecules which regulate important biological processes including blood clotting, formation of connective tissues, and functionality of secreted proteins, hormones, and signaling molecules. Sulfonation is ubiquitous in all tissues and widespread in nature (plants, animals, and microorganisms). Although sulfoconjugates were discovered over a century ago when, in 1875, Baumann isolated phenyl sulfate in the urine of a patient given phenol as an antiseptic, the significance of sulfonation and its roles in human diseases have been underappreciated until recent years. Here, we provide a current overview of the significance of sulfonation reactions in a variety of biological functions and medical conditions (with emphasis on cancer). We also discuss research areas that warrant further attention if we are to fully understand how deficiencies in sulfonation could impact human health which, in turn, could help define treatments to effect improvements in health.

## BACKGROUND

Our research team recently completed a genome-wide siRNA screen to identify genes that, when silenced, would significantly enhance the cytotoxic effects of cisplatin when added at a sub-lethal dose [1, 2]. This screen revealed a gene that when silenced increased the activity of platinum-based cytotoxic drugs (e.g. cisplatin, carboplatin, and oxaliplatin) as well as radiation and topoisomerase I inhibitors (e.g. irinotecan and topotecan) but not topoisomerase II inhibitors (e.g. doxorubicin). The gene encodes for 3′-phospoadenosine 5′-phosphosulfate (PAPS) synthase 1 (PAPSS1), a dual function enzyme comprising an ATP sulfurylase and a kinase domain. PAPSS1 functions sequentially to synthesize the biologically active form of sulfate (PAPS); the substrate used for sulfonation reactions in cells. PAPSS1 inhibition in combination with low-dose (IC_10_) cisplatin resulted in increased DNA damage, apoptosis and G1/S cell cycle arrest. At the IC_10_ of cisplatin (i.e. the dose of cisplatin that would cause 10% cell death), PAPSS1 inhibition reduced long-term viability of some cancer cells by 99% compared to non-targeting controls. These results suggest that sulfonation reactions are important for cancer cell survival. when attempting to understand what the role of PAPSS1 is in cancer we recognized a dearth of information. This review was undertaken to gain a better understanding of how sulfonation influences disease development and progression, with a particular focus on cancer.

**Figure 1:**
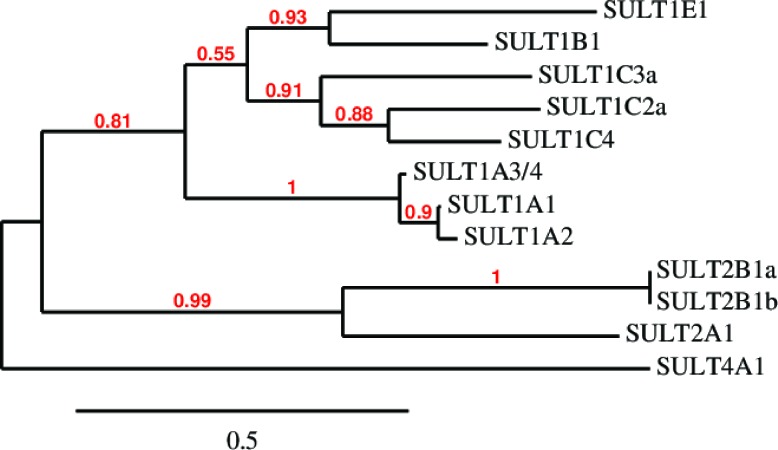
Amino acid sequence clustering of existing human SULTs Amino acid sequences were retrieved from the NCBI Protein database and phylogenetic analysis was performed using MABL. The branch support values are provided in red.

## EVOLUTION OF SULFONATION

**Figure 2:**
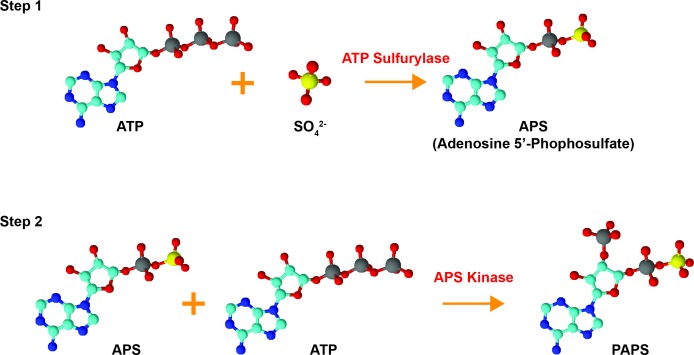
Bioactivation of inorganic sulfate Inorganic sulfate is converted to the biologically active form PAPS (3′-phosphoadenosine 5′-phosphosulfate) by the dual-function enzyme PAPSS. Inorganic sulfate is first converted to adenosine 5′-phosphosulfate (APS) by ATP sulfurylase. The intermediate molecule APS is subsequently phosphorylated *via* the APS kinase domain of PAPSS to form PAPS. The structures were drawn using ChemSketch and are color-coded as follows: red = oxygen, cyan = carbon, blue = nitrogen, yellow = sulfur, grey = phosphorous.

**Figure 3:**
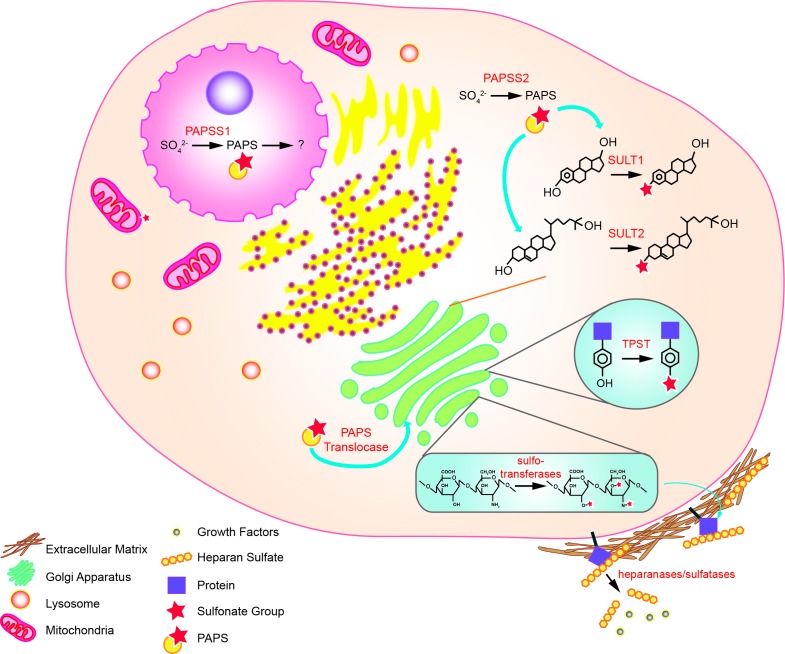
Sulfonation reactions in human cells In the nucleus, conversion of inorganic sulfate to PAPS is catalyzed by PAPSS1. In the cytoplasm, the same reaction is catalyzed by PAPSS2. The PAPS produced in the cytoplasm is used by cytosolic sulfotransferases to biotransform endo- and xeno-biotics. Cytosolic PAPS can also be transported to the golgi apparatus *via* the PAPS translocase, where tyrosine sulfation of proteins and sulfo-conjugation of polysaccharides occur. Sulfonated molecules such as heparan sulfates (HSs) may be secreted to the extracellular matrix or attached to cell surface proteins. Some of the HS may sequester growth factors that are released upon cleavage by heparanases and sulfatases.

Sulfonation plays an essential role in the biotransformation of endogenous compounds such as hormones and neurotransmitters as well as xenobiotics. Sulfonation reactions, catalyzed by sulfotransferases, involve the transfer of a sulfonate group (SO3-) from the obligate sulfate donor 3′-phoasphoadenosine-5′-phosphosulfate (PAPS) to a hydroxyl or an amino group [3–5]. In humans, PAPS is the biologically active form of sulfate; biosynthesized by the enzyme 3′-phosphoadenosine-5′-phosphosulfate synthase (PAPSS) [4]. The synthesis of PAPS involves two reactions: inorganic sulfate is first converted to adenosine-5′-phosphosulfate (APS) by ATP sulfurylase (EC 2.7.7.4) and this intermediate molecule is then phosphorylated by the APS kinase (EC 2.7.1.25) to form PAPS [6–8] (Figure 2). In prokaryotes, fungi, and plants, synthesis of PAPS is performed by two separate enzymes [9–13]. In animals, however, the ATP sulfurylase and the APS kinase are encoded by the same gene and translated into a single polypeptide which forms the dual-function enzyme PAPSS [6, 14]. Both APS and PAPS are activated sulfuryl donors that possess a phosphor-sulfate anhydride bond [14]. Phototrophic bacteria, algae, and some plants are known to utilize APS for the synthesis of the sulfur-containing amino acids cysteine and methionine via the assimilatory sulfate reduction pathway while chemotrophic bacteria, fungi, and some higher plants use PAPS [14–16]. The specificity for APS or PAPS is dependent on the presence of an iron-sulfur cluster in the sulfate-reducing enzymes of the organism [16]. Interestingly, these sulfate reduction pathways are not present in humans and other animals, meaning that methionine is an essential amino acid that can only be obtained through dietary sources [14, 16]. PAPS in animals is used for a variety of sulfonation reactions (summarized in Figure 3) including the biotransformation of endo- and xeno-biotics, as described below. Hence, PAPSS has evolved structurally and functionally from prokaryotes to multi-cellular eukaryotes.

In humans, PAPSS exists in two isoforms: PAPSS1 and PAPSS2 [17]. While both isoforms are expressed ubiquitously, they differ in cellular localization and tissue distribution. PAPSS1 is localized to the nucleus while PAPSS2 is found primarily in the cytoplasm [18, 19]. PAPSS1 is required for the re-localization of PAPSS2 from the cytoplasm to the nucleus for additional PAPS production [19]. In terms of tissue distribution, PAPSS1 is the predominantly expressed isoform in brain and skin while PAPSS2 is most expressed in the liver, cartilage, and adrenal glands [14]. Relative distribution of the two isoforms varies in other tissues [14]. Deficiencies in the two isoforms are associated with different medical conditions as discussed below [20]. Altogether, these findings support that while both PAPSS1 and PAPSS2 catalyze the production of the obligate sulfonate donor PAPS, the two isoforms have non-redundant functions.

## SULFOTRANSFERASES AND XENOBIOTIC METABOLISM

Sulfonation is most commonly known to be associated with the metabolism of xenobiotics that inactivate drugs such as acetaminophen by increasing their water solubility for excretion and decreasing their membrane permeability and biological activity through the addition of a charged moiety [5]. This modification is also partially responsible for drug resistance to chemotherapy in cancer treatments [21]. While PAPSS1 and PAPSS2 are responsible for the bioactivation of sulfate, sulfo-conjugation reactions are catalyzed by enzymes known as sulfotransferases [4, 5]. Sulfotransferases are mainly divided into two groups: they are either cytosolic or membrane-bound [5, 22]. Cytosolic sulfotransferases constitute the superfamily of enzymes known as SULTs which are involved in the sulfonation of xenobiotics and small endogenous compounds such as neurotransmitters and hormones. The membrane-bound sulfotransferases are found in the Golgi apparatus and are responsible for post-translational sulfonation of endogenous macromolecules such as proteins, lipids, and glycosaminoglycans [22–24]. Currently, 12 SULT isoforms have been identified and detected in human tissues [25, 26]. The Phylogenetic analysis of these isoforms was performed using MABL [27–33] and is shown based on amino sequence similarity in Figure 1. The chromosomal localization and tissue distribution of these isoforms have been described by Tibbs *et al*. in a recent review article [26]. Additional isoforms and splice variants have been predicted by other groups but are yet to be detected in human tissues at the protein level [26, 34, 35]. Generally, SULTs are grouped into four different families (SULT1, 2, 4, and 6) and they differ in their substrate specificity and tissue distribution [4, 25, 36]. SULTs are expressed at high levels during fetal development in humans [37]. In fact, some isoforms are only or primarily expressed during the prenatal period. The localization, expression levels, and the substrates (endogenous and foreign) of each SULT isoform characterized thus far are described in several comprehensive reviews [5, 37, 38]. A recent review by Coughtrie describes the function and organization of the different human SULT families [39]. SULT1 enzymes catalyze the sulfonation of catechnolamines and many other compounds [39]. SULT1A1 and SULT1B1 are the primary enzymes involved in the metabolism of xenobiotics in humans, making up nearly 70% of hepatic sulfotransferases. SULT1A1 is considered the major SULT isoform in human tissues as it is highly expressed in the liver and the gastrointestinal tract, conjugating small phenolic compounds such as estrogens, phytoestrogens, and minoxidil [40]. There are three isoforms within the SULT1A subfamily which differ in substrate specificity and thermostability despite sharing >90% sequence identity [41]. SULT2 enzymes are selective for steroids such as cholesterol and bile acids [5, 25, 42, 43]. The physiological functions of SULT4 and SULT6 are poorly understood [25, 42].

Sulfonation has been recognized as a high-affinity, low-capacity conjugation system that relies heavily on the availability of PAPS, which is dependent on its synthesis, use, and degradation [7, 26]. PAPS levels vary in different tissues and are believed to limit the sulfonation capacity of various cell types [26]. Although each SULT isoform is known to have different tissue distribution and affinity for specific classes of substrates, it is also believed that PAPS concentrations are important in regulating substrate selectivity. Interesting studies by Cook et al. suggest that nucleotide binding triggers a gating mechanism that affects substrate selectivity [44]. Following nucleotide binding, there is a conformational change in the protein that limits substrate access to the catalytic domain. The authors showed *in silico* predictions of how saturating concentrations of PAPS could substantially decrease the affinity of SULT2A1 for large substrates. Whether these gating mechanisms apply to all human SULTs and the exact mechanism of” how substrate selectivity is regulated in each isoform remain”s” to be investigated.

In most cases, sulfoconjugation is associated with detoxification. Some compounds, however, are bioactivated upon sulfonation by sulfotransferases [5]. Sulfonation could result in the generation of reactive electrophiles that can bind DNA, eliciting a mutagenic or even carcinogenic response [23, 36]. Based on screens conducted using recombinant bacteria, mammalian cell lines, and cell-free systems, about 100 compounds were identified to be genotoxic upon sulfonation by SULTs [36, 45]. Many studies have also shown that brachymorphic mice, which suffer from a PAPSS2 genetic defect (like patients with Pakistani spondyloepimetaphyseal dysplasia), are more resistant to tumorigenesis when exposed to procarcinogens known to be activated via sulfonation [46]. It is also possible that sulfo-conjugation may enhance the therapeutic activity of certain drugs. Minoxidil, an anti-hypertensive and hair growth-stimulating drug, is one such example where the sulfate metabolite is responsible for its biological activity [23, 47, 48].

## TYROSINE SULFONATION

Aside from biotransformation of xenobiotics, sulfonation is also the most abundant post-translational modification of tyrosine residues [49]. About 1% of the tyrosines are sulfonated [50]. While sulfonation is the appropriate term to describe the transfer of sulfonate groups (SO_3_), the same reaction has been described widely as sulfation in the literature, particularly when discussing proteins and proteoglycans that have been sulfonated (ie. tyrosine-sulfated proteins and heparan sulfates). Here, we will use the term “sulfates” when referring to sulfonated tyrosine residues and glycosaminoglycans as these structures are commonly referred to as sulfates in the literature.

Currently, there is no evidence of protein tyrosine sulfonation (PTS) in yeasts and in prokaryotes, suggesting that PTS first appeared in multicellular eurkaryotes [50, 51]. Tyrosylprotein sulfotransferase (TPST), the enzyme responsible for PTS, resides in the *trans* Golgi, and PAPS, the obligate substrate for the reaction, is transported from the cytoplasm to the Golgi via a PAPS translocase [50, 52–54]. PTS has a multitude of biological functions, many of which are still being characterized [22]. PTS can be important for the biological activity of certain neuropeptides. For instance, sulfonated cholescystokinin, a hormone important for the secretion of digestive enzymes, is at least 200 times more active than its unsulfonated counterpart [50]. Translated proteins can also be modified by PTS to diversify their functionality. As an example, gastrin normally regulates the secretion of gastric acid. When sulfonated by PTS, gastrin can also function as a pancreatic secretagog [55]. The extent of proteolytic processing of gastrin is also associated with PTS, suggesting that PTS can regulate proteolytic cleavage [50].

With increasing interests in the biological roles of PTS, studies have started to focus on the importance of PTS in mediating the immune response [51, 56–58]. At sites of inflammation, adhesion of leukocytes to activated endothelium requires interactions between P-selectin on the endothelial cells and P-selectin glycoprotein ligand (PSGL)-1 on leukocytes. PTS is necessary at the tyrosine residues of PSGL-1 to facilitate this interaction [51, 56–58]. The presence of sulfonated tyrosine residues is also proving essential for proper blood clotting in response to vessel injuries as well as binding of chemokines to the chemokine receptors CCR5 and CXCR4 [51, 59].

## HEPARAN SULFATES

Heparan sulfate (HS) is a polysaccharide that is produced by virtually all cells [60]. HSs are often attached to proteins forming heparin sulfate proteoglycans (HSPGs) at the cell surface or in the extracellular matrix (ECM) [61]. Its basic structure consists of alternating hexuronic acid (D-glucuronic acid (GlcA) or L-iduronic acid (IdoA)) and D-glucosamine (GlcN) units [62]. The carbohydrate backbone is constructed as a polymer and then modified by a series of enzymes including glycosyltransferases, O-sulfotransferases, and an epimerase in the Golgi apparatus to form the final structure [60, 61]. HSPGs are known to be structurally diverse, with great variability in their chain lengths and sulfation patterns. This gives rise to an immense number of HS species that can bind different proteins such as chemokines, growth factors, and enzymes and serve a variety of functions including immobilization, protection from proteolytic cleavage, as well as roles in embryonic development, angiogenesis, cell adhesion, blood coagulation, and lipid metabolism [61–64]. The biosynthesis and roles of HS can be further explored in depth in excellent previously published review articles [60, 61, 65–68].

## SULFONATION IN THE CONTEXT OF DISEASES AND CANCER

### Genetic defects and deregulation in the sulfonation pathway

Genetic defects in the sulfonation pathway can have a wide range of effects. For instance, mutations in the diastrophic dysplasia sulfate transporter are associated with a lethal autosomal recessive disorder called achondrogenesis type 1B. This disorder is characterized by short limbs and pulmonary hypoplasia due to abnormal skeletal development [69–71]. Loss-of-function mutations in PAPSS2 are associated with a type of dwarfism called brachyolmia type 4, a non-lethal genetic disorder that affects the spine [72]. Some, PAPSS2 mutations can lead to brachyolmia or more severe skeletal disorders such as spondyloepimetaphyseal dysplasia Pakisani type, characterized by a number of abnormalities in the skeleton and the cartilage between long bones resulting in short stature and bowed legs. [72].

In the context of tyrosine sulfation, there are two known human isoforms of TPST [73]. In mice, double knockouts of the two TPSTs result in post-natal pulmonary failure and death as the lungs fail to expand [74]. Loss of TPST-2 activity causes hypothyroidism in mice, suggesting that the two isoforms have non-redundant substrate specificities and that tyrosine sulfation is necessary for normal pulmonary and thyroid gland functions [74, 75].

As mentioned above, PTS is important for inflammatory response. Specifically, in conditions associated with airway inflammation such as asthma and chronic obstructive pulmonary disease (COPD), PTS is prevalent [76]. It is known that binding of chemokines to chemokine receptors is essential in the regulation of leukocyte trafficking [77, 78]. Studies have demonstrated that the affinity of chemokine receptors to different chemokines is dependent on the sulfonation states of the tyrosine residues on the chemokine receptors [77]. In COPD patients, PSGL-1 is up-regulated on the surface of all leukocyte populations, where PTS plays a critical role in enhancing the interaction between immune cells and the bronchial endothelium [78].

### Roles of sulfonation in viral infections

The cellular sulfonation pathway is also known to be important for viral infections [79, 80]. HS is ubiquitous on the surfaces of cells and the highly sulfonated nature of HS provides ample negative charges that could interact with the positively charged viral proteins, promoting initial interactions between viruses and host cells [80]. In some cases, the interaction is much more specific. For instance, herpes simplex virus type 1 (HSV-1) binds to HSs on target cells via envelope glycoproteins gB and gC, but viral entry is mediated through the interaction between viral glycoprotein D and a specific 3-O-sulfonated HS [80–83].

To date, HSs are known to be involved in at least 16 different types of viral infections, including hepatitis C, human papillomavirus (HPV) and human immunodeficiency virus (HIV) [80]. In the case of HIV, the sulfonation pathway appears to be critical in multiple steps of viral infection. It has been established the binding of HIV involves an interaction between the envelope glycoprotein gp120 and syndecans, which are transmembrane HSPGs found on the cell surfaces of T-cells and macrophages [80, 84]. While HIV-1 only infects CD4+ cells, the attachment of the virus to the HSs of a non-permissive cell actually aids the virus in retaining its infectivity for a longer period than it would otherwise as a free virus [85]. This suggests that cells lacking CD4 expression may provide a reservoir for any bound HIV-1 [85].

Successful infection of HIV-1 virus requires expression of CD4 as well as the presence of specific co-receptors on the host cells. The chemokine receptor CCR5 is a major co-receptor that facilitates the entry of HIV-1 into target cells [59]. CXCR4 is another chemokine receptor that is commonly used by HIV-1 viruses as the infection progresses [86]. Based on studies completed by Farzan et al., both CCR5 and CXCR4 are sulfonated [59]. Specifically, when the tyrosine residues at the N terminus of CCR5 are mutated to phenylalanine and therefore are not sulfonated, there is a marked decrease in the binding of CCR5 with MIP-1α, MIP-1β, and gp120/CD4 complexes, significantly reducing the ability of HIV-1 to enter their target cells [59]. It has also been recently established that PAPSS1 plays a critical role in gene expression from the long terminal repeat promoter following provirus establishment [79]. In this example, the transcriptional activity may be influenced by sulfonation in an epigenetic manner [79]. Although the mechanism is not fully understood, it is clear from the work of Bruce *et al*. that sulfonation in the nucleus is required for proper expression of LTR-driven genes which is needed for viral replication [79]. Finally, HIV-1-infected cells are known to release a protein called Tat (transactivator protein), the causative agent of AIDS (acquired immune deficiency syndrome), which is known to damage tissues and cells and is associated with neurotoxicity and increased risks of developing cancer [80, 87, 88]. Tat can also cause non-permissive cells to become susceptible to HIV infections [80]. The internalization of Tat into cells is facilitated, again, by HSs [80, 88]. As summarized here using HIV as an example, sulfonation plays essential roles in viruses' ability to infect and complete their life cycles in humans. Also the structural diversity of HS is immense and extensive studies in this area are therefore necessary to fully understand how specific HSs aid in the infections of different viruses.

### Sulfonation and cancer

While phosphorylation is extensively studied in cancer development and treatments, sulfonation has been largely overlooked in the context of oncology [89]. It is only in recent years that there is a growing body of evidence that individual differences in various genes of the sulfonation pathway may contribute to carcinogenesis and patient survival. For instance, polymorphisms in SULT1E1, a sulfotransferase that is involved in estrogen metabolism, are correlated with the survival of patients with estrogen-dependent cancers. Studies conducted by Hirata et al. and Rebbeck *et al*. demonstrated that polymorphisms in SULT1E1 are associated with greater endometrial cancer risks [90, 91]. In estrogen receptor (ER) positive breast cancers, tumorigenesis and disease progression rely on the presence of estrogen [92]. SULT1E1 is known to be overexpressed (relative to breast cancer cells) in normal human mammary epithelial cells [93]. In a recent study, Xu et al. demonstrated that overexpression of SULT1E1 and PAPSS1 can block estrogen-stimulated cell proliferation in MCF-7 breast cancer cells, while promoting apoptosis through upregulation of the pro-apoptotic gene *bax* [94]. It is important to note that the role of estrogen sulfonation in cancer patients could be complicated as some SULT isoforms have overlapping substrate specificity and need to be considered. For instance, SULT1A1 catalyzes the sulfonation of a variety of small phenolic compounds including estrogen and is known to be associated with breast cancer risks [95, 96]. Tamoxifen, an agent that is commonly used to treat breast cancer, is a prodrug that is metabolized by SULT1A1 to its activated metabolite 4-hydroxytamoxifen (4-OH TAM) in the liver. Polymorphisms in SULT1A1 are known to exist and one particular variant SULT1A1*2 (where Arg at codon 213 is substituted with His) has been seen in some breast cancer patients [97]. This enzyme has significantly lower enzymatic activity and thermostability than the wild-type enzyme [97]. While the investigators expected SULT1A1*2 to correlate with improved survival due to reduced sulfonation of 4-OH TAM they surprisingly found that the variant allele was associated with poor survival in patients who were treated with tamoxifen [97]. This observation was supported by another study conducted by Wegman et al [98]. It was suggested that the sulfated 4-OH TAM metabolite actually serves as a potent inducer of apoptosis, thus improving the survival of individuals with the SULT1A1 genotype that has higher enzymatic activity [99]. In another study, women bearing benign and malignant gynecological tumors were found to have a higher frequency of the common allele of SULT1A1, suggesting that there was an increase in endometrial cancer risk with greater SULT1A1 activity [100]. However, other studies have found either no or negative correlation between SULT1A1*2 and endometrial cancer risks. Clearly there is a need for further investigations [101–103].

Polymorphisms in SULT1A1 have also been studied in patients with estrogen-independent cancers. Several studies in lung cancer have demonstrated that the variant SULT1A1*2 allele is associated with increased risks of lung cancer, especially for smokers [104–106]. Interestingly, contrasting results were found in bladder cancer. Women and never smokers with the His (213) allele were found to have reduced risks of bladder cancer [107]. The variant allele even appears to provide some protective effects for smokers against bladder cancer [107, 108]. The same polymorphism is associated with significantly greater risks of upper urinary tract urothelial cell carcinoma, head and neck cancer, gastric cancer, and colorectal cancer, particularly in smokers and consumers of alcohol and red meat [109–112]. These findings suggest that SULT1A1 activity plays roles in carcinogenesis in a tissue-specific manner.

Aside from SULTs, heparan sulfate proteoglycans and chondroitin sulfate proteoglycans (CSPGs) have also been shown to be associated with cancer. Chondroitin sulfates (CS) are sulfonated glycosaminoglycans complexed with core proteins to form CSPGs that reside at the cell surface and extracellular matrix [113]. CSs are synthesized during embryonic development as polysaccharides of alternating D-glucuronic acid and D-Nacetyl-galactosamine units which are further modified via sulfonation [113, 114]. CSPGs are highly expressed in the vessels of brain tumors and the stroma of various types of cancer including melanoma, prostate, breast, testicular, colon, pancreatic, and gastric cancers and are known to play important roles in tumor growth and invasion [113–118]. The anionic nature of CS chains facilitates interactions between cells through binding of ligands and receptors that result in activation of signaling pathways that promote tumor growth and metastasis [113, 119, 120]. In head and neck cancer, patients appear to excrete chondroitin sulfates in their urine, which could be useful for diagnostic purposes [114]. Recently, Poh et al. have synthesized a library of CS disaccharides to evaluate the effects of different sulfonation patterns on breast cancer cell viability [121]. Their results suggest that the presence of specific sulfonation patterns could lead to growth inhibition in a triple negative breast cancer cell line. A review written by Asimakopoulou *et al*. describes additional biological roles played by CS in different types of malignancies as well as an overview of several modified CSs that have been tested as targeting and therapeutic agents [113].

Similar to CSPGs, the abundance and diversity of HSPGs at normal and tumor cell surfaces and in the extracellular matrix affect cancer biology by initiating transformation of normal cells, modulating tumor growth, and promoting metastasis [122]. For instance, Glypican-3 (GPC3) is a HSPG that negatively regulates cell proliferation and survival [123]. Several studies have shown that GPC3 expression is reduced in mesothelioma, breast cancer, and ovarian cancer cells [122–125]. In other studies, induction of the sulfatase SULF2 was found in breast cancer and lung cancer [126, 127]. Sulf-2 expression is associated with activation of the Wnt signaling pathway which promotes cell proliferation [126, 128]. In contrast, suppression of Sulf-2 in cancer cells inhibited cell growth and even partially reversed transformation *in vitro* [126].

As mentioned earlier, different sulfonation patterns on HSPGs enable them to bind different molecules including growth factors. HSPGs therefore can sequester growth factors and release them upon degradation of their HS chains by heparanases or changes in sulfonation patterns by sulfatases, directly regulating cell proliferation [89, 122]. Vascular endothelial growth factor (VEGF) and fibroblast growth factors (FGF) are growth factors that are also released from HSPGs by endosulfatases and heparanases in the extracellular matrix [89, 129]. Pericellular HSPGs can facilitate the binding of VEGF and FGF with their corresponding receptors on endothelial cells and activate these signaling pathways that are central to the process of angiogenesis [130]. While HSPGs can sequester or release pro-angiogenic factors, other HSPGs can inhibit angiogenesis by binding the anti-angiogenic factor endostatin. The ultimate stimulation or inhibition of angiogenesis is therefore a fine balance between the concentrations and binding affinities of pro- and anti-angiogenic factors, which are highly dependent on the HSPG profiles of cells in the tumor microenvironment [122, 130]. As an example, Cole *et al*. have demonstrated that down-regulation of HS 6-O-sulfotransferases 1 and 2 leads to reduced 6-O-sulfonation, reduced endothelial cell signaling and angiogenesis, and significantly delays the growth of ovarian cancer tumours *in vivo* [131]. A recent study conducted by Mao *et al*. reveals the role of the heparan sulfate sulfotransferase 3-OST3A (HS3ST3A) in regulating tumour growth in breast cancer by controlling the tumour microenvironment [132]. Depending on the tumour subtype, 3-OST3A expression could induce onocgenic or tumour-suppressive effects and even affect therapeutic responses by regulating the activity of FGFs. A similar study was conducted by Kumar *et al*., investigating the role of heparan sulfate 3-O-sulfotransferase 2 (HS3ST2) on the invasiveness of highly and low invasive breast cancer cells [133]. While FGFs are more commonly known to be involved in cancer cell proliferation, Jung *et al*. has recently shown that PAPSS2 depletion leads to under-sulfonation of HSPGs, which in turn augments FGFR1 and Akt signaling, ultimately inducing premature cellular senescence, a tumour-suppressive mechanism that could lead to tumour clearance through an innate immune response [134]. In terms of tumor metastasis, HSPGs, again, play opposing roles. Along with collagen and laminin, HSPGs construct a protective barrier through tight cell-cell and cell-ECM adhesions [135]. Heparanases and other enzymes released by tumor cells help to modify these barriers as needed for invasion and metastasis [136–139]. Therefore, in most cases, tumor cells with low levels of HSPGs are correlated with high metastatic potential [135]. Specifically, studies in recent years have demonstrated that the expression of a specific HSPG, syndecan-1, is associated with changes in tumor cell morphology. Syndecan-1 expression is negatively correlated with metastatic potential and poor prognosis in many different types of malignancies including mesothelioma, colon, lung, liver, breast, and head and neck cancer [135, 140–145]. The involvement of syndecan-1 in metastasis is explored in depth in a review written by Sanderson [135]. Since the interactions between tumour cells and the endothelium during metastasis are poorly understood, Martinez *et al*. conducted a study and demonstrated that cancer-associated glycosaminoglycans can serve as ligands for selectins in the endothelium and are recognized and bound based on sulfonation density and pH conditions [146]. These interactions help promote distant metastases.

While cell surface HSPGs are important for preventing tumor invasion, HSPGs in the ECM are involved in promoting metastasis. Extracellular HSPGs secreted by cells are known as perlecans while syndecans are cell surface HSPGs that could also be shed either into the ECM or into blood [135]. Studies have shown that suppression of perlecans decreases the invasiveness of metastatic melanoma and prostate cancer cells [147, 148]. While the mechanism of action of perlecans is yet to be defined, it has been suggested that perlecans and other HSPGs in the ECM may bind chemokines and growth factors, establishing gradients that direct the motility of migrating tumor cells [135].

As described above, HSPGs form a physical barrier with collagen to prevent invasion. Soluble HSPGs could also promote tumor cell invasion by interfering with cell-cell interactions. Studies suggest that exogenous HSs allow non-invasive cells to become highly invasive in several rodent tumor and human leukemia cell lines [149, 150]. Once tumor cells have escaped from their primary site into the vasculature, their cell surface HSPGs may aid in their motility as well as their extravasation out of circulation [135]. For this reason, intravenous administrations of different species of HSs and anti-HSPG antibodies were tested in an attempt to block HS function in the vasculature and ultimately inhibit metastases [151–153].

In summary, tumor cells may secrete heparanases and other enzymes to degrade cell-surface HSPGs and ECM-bound HSPGs in the proximity to initiate the metastatic processes. Subsequently, cell surface HSPGs aid in migration, extravasation: promoting “seeding” at their metastatic sites. All of these steps involve a variety of HSPG species, enzymes, and signaling molecules that are dynamically regulated by the tumor cells. This suggests great opportunities for the development of therapeutics, targeting HSPGs including the use of modified HSPGs, antibodies, and heparin. Heparin, an anticoagulant, is structurally similar to HSPGs and has been found to be anti-metastatic in multiple animal models for undefined reasons. These strategies are discussed in detail in several review articles [122, 153–156]. While the connection between HSPGs and cancer is well-established, there is still much to learn because the role is complex, depending on the tissue type, tumor subtype, the presence of enzymes, growth factors, and other HSPGs, as well as the localization (cell-surface vs. ECM) and structural characteristics (sulfonation pattern) of each HSPG. It is also important to explore how the activity of PAPSS enzymes and the availability of PAPS, which are utilized in all sulfonation reactions, affect the structure and function of HSPGs.

## SULFONATION IN THE NUCLEUS: THE UNCHARTED TERRITORY

Sulfonation is known to be involved in a broad spectrum of biological processes in both healthy and diseased cells. As indicated above, most studies have focused on the importance of sulfonation in cell-cell or cell-ECM communications and the modification of endo- and xenobiotics. However, very little is known about the sulfonation pathway in the nucleus, the control center of the cell. As mentioned above, two isoforms of PAPSS exist in humans, both of which contain a nuclear localisation signal (NLS) for translocation from the cytoplasm to the nucleus of the cell [19]. The NLS is more efficient in PAPSS1 and subcellular localization studies conducted by Besset et al. have shown that PAPSS1 localizes to the nucleus while PAPSS2 is predominantly found in the cytoplasm [18]. When co-expressed with PAPSS1, PAPSS2 is relocated to the nucleus [18]. Therefore, PAPSS2 may relocate to the nucleus when a greater demand for PAPS needs to be met in the nucleus. These data suggest that there is a critically important role of the sulfonation pathway in the nucleus. The existence of a functional sulfonation pathway is further confirmed by studies completed by Bruce *et al*. where PAPSS1 was identified to be involved in suppressing transcriptional activities from the LTR promoter upon provirus establishment in the host cells [79].

**Figure 4:**
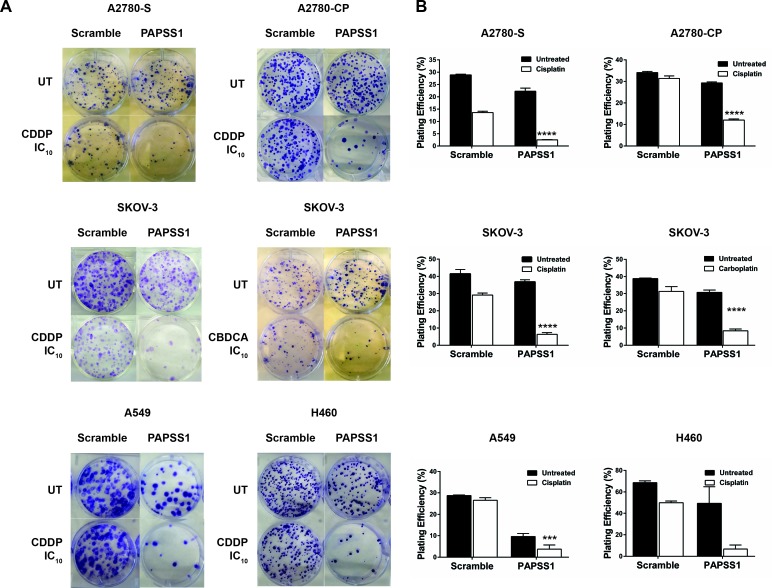
PAPSS1 knockdown sensitizes ovarian and lung cancer cells to platins Cisplatin (CDDP) sensitive (A2780-S) and resistant (A2780-CP) A2780, SKOV-3, A549, and H460 were seeded in 6-well plates and transfected with a pool of three PAPSS1-targeting or non-targeting siRNA duplexes the following day. The empirically determined IC10 of the corresponding platinum agent based on a 72h cell viability assay was added to the cells 24 hours post-transfection (0.112, 1.24, 0.98 μM CDDP for A2780-S, - CP, and SKOV-3 respectively; 19.3 μM carboplatin (CBDCA) for SKOV-3; 0.71 and 0.52 μM CDDP for A549 and H460, respectively). At 24 hours post-treatment, the cells were harvested and re-seeded for colony formation. The cells were subsequently incubated for 14 days undisturbed, after which the colonies were fixed and stained with 0.5% w/v crystal violet in 6.25% glutaraldehyde and counted. The plating efficiency (PE) was calculated using the equation (# colonies formed/#cells seeded) x100%. Representative images of the treatment conditions are shown in A and the PE values are plotted in B. All data are shown as mean ± SEM. Two-way ANOVA with Tukey adjustments for multiple tests comparison was used and the statistical significance of the sensitization effects of PAPSS1 knockdown is highlighted for each cell line (****p < 0.0001 for platin-treated scramble *vs*. PAPSS1-silenced cells).

In our laboratory, an siRNA screen led to PAPSS1 as a target that may potentiate non-small cell lung cancer (NSCLC) cells to cisplatin treatment [1]. Validation studies further demonstrated that PAPSS1 can be suppressed to enhance the therapeutic activity of a range of DNA damaging agents, including radiation, other platinum-based agents, as well as topoisomerase I inhibitors [1]. The sensitization effects were observed in both platinum-sensitive and resistant cell lines via colony formation assays (Figure 4) [1, 2]. This sensitization was selective for cancer cells as strong inhibition of PAPSS1 at the protein level did not sensitize normal bronchial epithelial cells to cisplatin treatment [1]. Validation of PAPSS1 as a therapeutic target has been further demonstrated in multi-cellular tumor spheroid models as well as in zebrafish and rodent models (Leung *et al*., submitted manuscript). These results suggest that the sulfonation pathway plays an important role in the survival of cancer cells following exposure to chemotherapy.

According to the cBioPortal database, only approximately 7% of breast cancers and 2% of lung adenocarcinoma harbour PAPSS1 amplifications and mutations [1]. Currently, there is no database that can assess how PAPSS1 expression affects treatment response. A study conducted by Shih *et al*. has shown that single nucleotide polymorphisms in the ATP sulfurylase domain of PAPSS1 was correlated with poor survival in patients with familial or early onset hepatocellular carcinoma (HCC) [157]. The causal relationships between PAPSS1 and HCC survival has not been elucidated and the mechanism(s) by which PAPSS1 sensitizes NSCLC and ovarian cancer cells to DNA damage are yet to be defined. While our previous studies demonstrated increased accumulation of DNA damage when PAPSS1-deficient NSCLC cells were treated with cisplatin and topotecan, further research is needed to fully understand the mechanism of action as there is a lack of knowledge of the roles of sulfonation in the nucleus of eukaryotic cells. Recent studies have shown that PAPSS1 can form a heterodimer with the evolutionarily older isoform PAPSS2 [158, 159]. However, the function of this heterodimer and its potential role in regulating overall PAPS production is unclear. Therefore, although the PAPSS enzymes are starting to become recognized as potential contributors to cancer and other human diseases, research in the two enzymes that produce the substrate for all cellular sulfonation reactions is in its infancy and warrants further attention.

## CONCLUSIONS AND FUTURE PERSPECTIVES

Sulfonation is a key post-translational modification process that affects a tremendous number of biological activities through cell-cell and cell-matrix communication. There is a great amount of research concerning the roles of glycosaminoglycans and proteglycans in diseases but it has been difficult to apply this knowledge to therapeutic applications because of the structural diversity and the tissue-specific nature of responses. There are growing interests in the roles of sulfotransferases in different types of malignancies; however, conflicting results suggest the need for greater sample sizes and more studies to understand how sulfotransferase activities affect cancer development, progression, and treatment responses in different organs. Protein tyrosine sulfonation is another area that is largely underexplored. Which proteins in the proteome are tyrosine sulfonated? Part of this challenge concerns development of reliable methods for measuring sulfonated tyrosines on proteins. Some groups have been trying to predict sites of PTS by developing software and algorithms while more and more tyrosine sulfonated proteins are being identified in recent years [51, 160]. We are, however, still far from finding all tyrosine sulfonations and this, in turn, limits our ability to understand the role that tyrosine sulfonation plays in biology. Other questions that will need to be addressed in the future include: 1) How do cells alter HSPG expression on the cell surface? 2) How does the supply of PAPS affect the sulfonation efficiency of different sulfotransferases in different tissues? 3) Which processes require PAPS in the nucleus? From our perspective, focused on cancer, we believe that answering these questions will first require the recognition that sulfonated proteins and glycans in different cellular compartments and organs play important roles in cancer cell proliferation and survival.

Currently, a great amount of effort is focused on discovering inhibitors that target phosphorylated proteins to reduce signaling of hyper-activated pathways in cancer cells. There are, however, still many unexplored areas that could be of great importance in cancer treatment. Sulfonation is one where many correlations with tumorigenesis and patient survival have been recognized, but little is known about the mechanisms of action and casual relationships. The area is extremely broad and requires extensive research looking at the impact of sulfonation in various cellular compartments, tissue types, as well as its involvement in cancer cell mobility, invasion, and metastasis. Death from cancer is almost always attributed to metastasis [161] and there is a need to explore all potential strategies that may prevent or slow the development of metastasis. In this context, more research is needed to understand the role of PAPSS enzymes in cancer cell biology.
